# Exploring Graph Theory Mechanisms of Fluid Intelligence in the DLPFC: Insights From Resting‐State fNIRS Across Various Time Windows

**DOI:** 10.1002/brb3.70386

**Published:** 2025-02-28

**Authors:** Yuemeng Wang, Zhencai Chen, Ziqi Cai, Wenqun Ao, Qi Li, Ming Xu, Suyun Zhou

**Affiliations:** ^1^ Key Laboratory of Psychology of TCM and Brain Science, Jiangxi Administration of Traditional Chinese Medicine Jiangxi University of Chinese Medicine Nanchang Jiangxi province China; ^2^ Department of Psychology Jiangxi University of Chinese Medicine Nanchang Jiangxi province China; ^3^ Key Laboratory of Emotional Disorders Detection and Rehabilitation, Jiangxi Provincial Department of Education Jiangxi University of Chinese Medicine Nanchang Jiangxi province China

**Keywords:** DLPFC, fluid intelligence, resting‐state fNIRS, time‐windowed graph theory

## Abstract

**Background:**

Brain imaging technologies can measure fluid intelligence (gF) levels more directly, objectively, and dynamically, compared to traditional questionnaire scales. To clarify the temporal mechanisms of graph theory in measuring gF, this study investigated the relationship between graph theoretical indicators in the dorsolateral prefrontal cortex (DLPFC) and gF levels under various time windows.

**Methods:**

Using 30‐min resting‐state fNIRS (rs‐fNIRS) data and Raven's Advanced Progressive Matrices from 116 healthy participants, the relationship between individual gF levels and DLPFC brain signals was analyzed using average degree (AD) and global efficiency (Eglob).

**Results:**

AD and Eglob in the resting‐state DLPFC were significantly negatively correlated with the RAPM score. Considering the effectiveness and efficiency of gF measurement, a 2‐min data collection might suffice, while for Eglob, more than 15‐min collection was more effective.

**Conclusion:**

These findings help clarify brain indicators and demonstrate the effectiveness of rs‐fNIRS in intelligence measurement, providing a theoretical and practical basis for portable and objective gF assessment .

## Graphical abstract

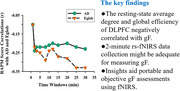

## 1 Introduction

Fluid intelligence (gF), a general ability encompassing multiple cognitive processes and involving various cortical regions across the whole brain, represents the capacity to reason and solve novel problems (Cattell 1971). Compared to questionnaire‐based assessments, brain imaging technologies (e.g., fNIRS, fMRI, EEG) offer a more direct, objective, and dynamic approach to measuring the brain functions associated with gF (Pinti et al. 2023; Yen et al. 2023). Among these technologies, the portability (Balardin et al. 2017; Kiguchi et al. 2012; Tran et al. 2023; Zhao et al. 2021), strong resistance to interference (Fishburn et al. 2014; Yang et al. 2022), and cost‐effectiveness (Kwasa et al. 2023; Rahman et al. 2020) of fNIRS technology, making it a promising tool for the standardized and objective measurement of gF. Brain imaging studies have confirmed that the functional connectivity (FC) of the frontal lobe reflects the state of gF (Chan et al. 2018; Cipolotti et al. 2023). However, further exploration of the graph theory mechanisms underlying gF based on fNIRS brain signals is necessary to provide both theoretical and practical foundations for developing portable and objective gF measurement methods.

Studies of resting‐state functional brain imaging for gF have shown that the level of resting‐state FC within the frontoparietal network can serve as a basis for predicting performance on complex tasks, with a positive correlation between intrinsic FC mean of the frontoparietal network and cognitive ability (Chenot et al. 2021; Greene et al. 2018). Another study, based on the analysis of global brain functional connectivity (GBC), found that the verbal‐analytic reasoning ability of gF is negatively correlated with the FC of the right inferior frontal gyrus (Chen et al. 2017). Although some studies suggest that the correlation between gF and cerebral FC might be a spurious association caused by head motion (Li et al. 2019; Siegel et al. 2017), the ability to maintain a stable resting state during the experiment (including minimizing head motion) may be linked to executive functions, which are inherently associated with gF (Panikratova et al. 2020; Santarnecchi et al. 2021). Therefore, the level of gF can, to some extent, be reflected and assessed by the functional connectivity of the frontoparietal network during the resting state. However, care should be taken to avoid overinterpreting the causal mechanisms underlying this relationship.

Meanwhile, in graph theory, average degree (AD) measures the average number of connections a node makes within a network, while global efficiency (Eglob) measures the efficiency of information transfer throughout the network (Latora and Marchiori 2001; J.‐H. Wang et al. 2011). These indicators provide insights into the importance of nodes in brain networks and the functional efficiency of the network. Some studies have shown that these indicators are correlated with gF, with higher gF scores associated with shorter path lengths and higher Eglob of brain networks (Langer et al. 2012; Li et al. 2009). Therefore, this study aims to explore the potential graph theory mechanisms of gF in the dorsolateral prefrontal cortex (DLPFC) through a correlation analysis of AD and Eglob of DLPFC channel connections with the Raven's Advanced Progressive Matrices (RAPM) score (Raven et al. 1998).

Additionally, most researches have shown that the duration of resting‐state fMRI scans can impact the reproducibility of results (Birn et al. 2013; Liao et al. 2013; Mejia et al. 2018; Teeuw et al. 2021; J.‐H. Wang et al. 2011; Y. Wang et al. 2021). In particular, the reliability of graph metrics may be influenced by scan length (Birn et al. 2013; Liao et al. 2013). A previous study found that resting‐state scans last 300 s or less tend to show poor test‐retest reliability of connectivity (Noble et al. 2017). Van Dijk et al. (2010) has examined conventional connectivity parameters obtained from resting‐state data and concluded that a 5‐min scan duration was adequate for obtaining reliable estimates. However, others have proposed that at least 10‐min of resting‐state data is necessary for good reliability (Birn et al. 2013). Specifically, 30‐min of resting‐state data almost doubled the reliability achieved with shorter scan durations (Finn et al. 2017; Shah et al. 2016).

Considering the influence of resting‐state fNIRS (rs‐fNIRS) data collection time on data and result stability, our present study analyzed the relationship between 30‐min graph‐theoretical indicators and their corresponding AD and Eglob under 11 time windows. Moreover, to further explore the efficacy of graph‐theoretical indicators associated with gF, this study also tries to identify the suitable time window for measuring gF using rs‐fNIRS.

All in all, the current study aims to investigate the association between RAPM and the AD level, Eglob of rs‐fNIRS DLPFC signal across various time windows, with the goal of preliminarily clarify graph‐theoretical indicators and suitable rs‐fNIRS acquisition time relevant to gF. Moreover, the effectiveness of rs‐fNIRS technology in gF measurement could be further verified, providing a theoretical and empirical basis for the development of portable and objective gF measurement. Additionally, based on the consistency between the results of the current rs‐fNIRS research results and the previous findings using fMRI technology, the discovery of gF measurement using fMRI technology may be transferred into practical applications via fNIRS technology.

## 2 Methods

### 2.1 Participants

A total of 152 healthy college students were recruited to participate in the experiment, including 88 females and 64 males, with an average age of 20.05 ± 2.01 years. The inclusion criteria were as follows: (1) age ≥ 18 years old; (2) no history of mental illness or psychological disorders; (3) no symptoms of discomfort; (4) no consumption of alcoholic or stimulant drinks, no use of hormonal drugs, and no staying up late or other disruptive behaviors in the 2 days preceding the experiment; and (5) no prior experience with the RAPM test.

The exclusion criteria for the analysis of fNIRS data were as follows: (1) The resting‐state data collection time in this study was set at 30 min. A total of 29 participants, could not maintain a resting state throughout the experiment (e.g., any kinds of opening eyes, talking, snoozing, or withdrawing midway due to fatigue or loss of interest), and were excluded. (2) Seven participants with missing (4 participants) or noisy channels (3 participants, coefficient of variation (CV) > 15%) were also excluded. Based on the above exclusion criteria, a total of 116 participants were included in the final analysis of fNIRS data. All participants signed a written informed consent form prior to the experiment and were provided with compensation after the experiment. This study was approved by the Institutional Review Board (IRB, LL‐2023C0108‐01) of the Department of Psychology, Jiangxi University of Chinese Medicine, China.

### 2.2 Experiment Procedure and Materials

The entire procedure consisted of two parts, including a rs‐fNIRS experiment and a paper‐and‐pencil test. First, the rs‐fNIRS experiment was conducted, the participants were required to close their eyes, remain still, and stay awake within 30‐min. Second, the RAPM was used to measure gF level. After the rs‐fNIRS experiment, participants completed the RAPM test which consists of set I (12 items) and set II (36 items). The set I was served as practice without timing, while the set II was the time‐limited test set, to be completed within 40 min (Raven et al. 1998).

### 2.3 fNIRS Data Acquisition

**FIGURE 1:**
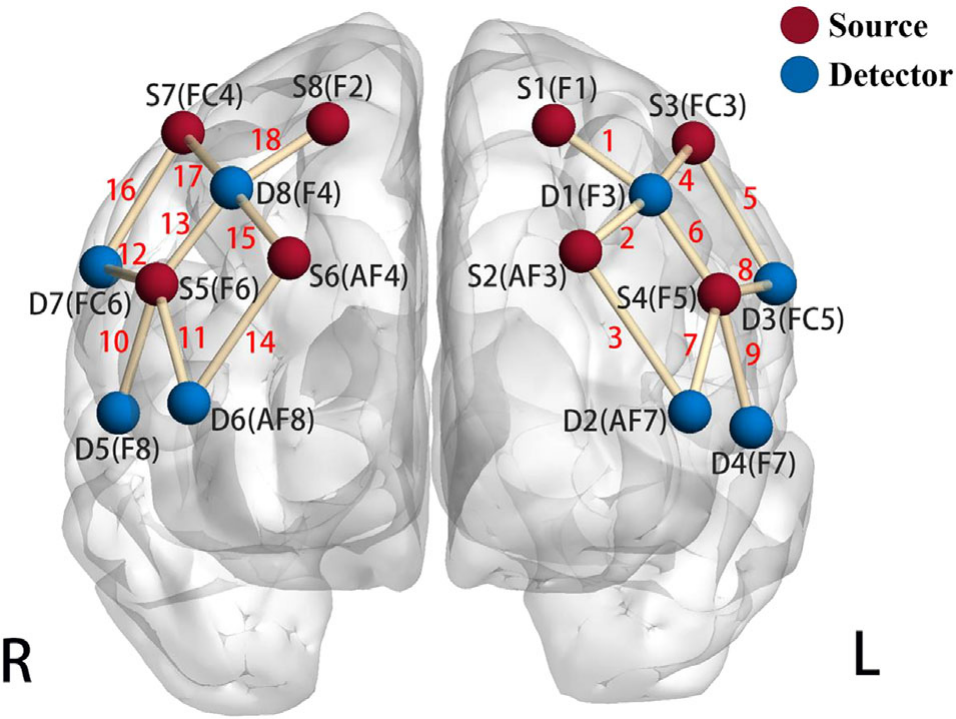
Optode and channel montage of the DLPFC. L and R represent the left and right hemispheres, respectively. This glass brain was generated using BrainNet Viewer (Xia et al. 2013).

The fNIRS signal was acquired with NIRSport 8 × 8 device and NIRStar15‐2 software (NIRX, Minneapolis, USA), which consisted of 8 LED sources (Source) and 8 APD detectors (Detector), generating 18 measurement channels (CH). The device was configured to detect deoxygenated and oxygenated hemoglobin with wavelengths of 760 nm and 850 nm. The Source and Detector optodes were placed on the scalp surface corresponding to the DLPFC according to the 10–20 international standard electrode system, as shown in the Figure 1 with their CHs and labels.

### 2.4 Data Analysis

#### 2.4.1 RAPM Descriptive Statistics

The RAPM score was calculated by summing the correct responses (1 for correct, 0 for incorrect) for the 36 items in the set II.

#### 2.4.2 Preprocessing of Rs‐fNIRS Data

The preprocessing of rs‐fNIRS data primarily involved the use of the Homer 3 toolbox (Huppert et al. 2009). First, the data was checked for NaN values caused by saturation, and the NaNs were Spline interpolated. Second, the motion artifacts were estimated with parameters set as tMotion = 0.5 s, tMask = 5 s, STDEVthresh = 15, and AMPthresh = 0.5. Subsequently, a combination of Spline interpolation and Wavelet transformation was applied to correct for motion artifacts (Di Lorenzo et al. 2019; Huang and Li 2024). Then, a bandpass filter with a frequency range of 0.01–0.08 Hz was applied to remove high‐frequency noise, baseline drift, and physiological interference (e.g., heart rate, respiration, Mayer wave components), reducing the influence of signals from superficial layers like the scalp (Pinti et al. 2019). Finally, the processed intensity was calculated based on the modified Beer–Lambert law (Kocsis et al. 2006), resulting in signals representing relative concentration changes in oxyhemoglobin (HbO), deoxyhemoglobin (HbR), and total hemoglobin (HbT). Since the HbO is the most sensitive indicator of regional cerebral blood flow changes in NIRS measurements (Hoshi 2007), it was used for subsequent analysis in this study.

To construct FC matrices for AD and Eglob calculations, custom scripts in MATLAB R2017b (The MathWorks Inc., Natick, MA) were used. Pearson correlation analysis was performed on the HbO signals from the 18 channels in the DLPFC. The resulting FC matrices were then subjected to Fisher's *r*‐to‐*Z* transformation to linearize the correlation coefficients, enhancing their suitability for subsequent graph‐theoretical analyses.

#### 2.4.3 AD and Eglob Calculations

AD refers to the average degrees across all nodes within a network and AD is frequently used as a measure of network density (Rubinov and Sporns 2010). Using custom MATLAB scripts, the mean FCs across all DLPFC channels were calculated as the AD. The formula for AD, Equation (1) shows that *N* represents the number of channels, and the degree of the ith node in the network is denoted as *c*_i_ (Stam and Reijneveld 2007):

(1) $$C=\frac{1}{N}\sum_{i=1}^{N}c_{i}$$

Eglob is the average inverse shortest path length, a measure of network connectivity that indicates the efficiency with which information is transferred in a network. A network with high Eglob indicates that information can be transmitted quickly and efficiently between nodes (Latora and Marchiori 2001). The calculation of Eglob for the DLPFC channel connectivity was performed at various binarization thresholds of FC matrices (range from 0.1 to 0.5 in steps of 0.1) using custom MATLAB scripts incorporating the efficiency_bin function from the Brain Connectivity Toolbox (Rubinov and Sporns 2010). The formula of Eglob, Equation (2), shows that *N* represents the number of channels, and *d_ij_* represents the shortest path length between two generic points *i* and *j* (Latora and Marchiori 2003):

(2) $$E\left(G\right)=\frac{1}{N(N-1)}\sum_{i,j\in G,i\neq j}\frac{1}{dij}$$

#### 2.4.4 Correlations Between RAPM Score and the AD, Eglob Across Various Time Windows

This study analyzed the gF relevant brain network indicators of DLPFC by using the AD and the Eglob of DLPFC channel FCs. Specifically, partial correlation analyses (controlling for age and sex) were conducted between the AD, the Eglob of DLPFC channel connectivity under the 30‐min time window and the RAPM score.

Additionally, to determine which time window of rs‐fNIRS acquisition was more suitable for measuring gF, this study divided the 30‐min data acquisition period into 11 different time windows for subsequent analyses (i.e., 0 to 1, 0 to 2, 0 to 3, 0 to 5, 0 to 8, 0 to 10, 0 to 12, 0 to 15, 0 to 20, 0 to 25, and 30‐min). First, to explore at which time window the graph theoretical indicators begin to stabilize, the mean values of AD and Eglob were calculated for 116 participants across the various time windows. Second, the AD and Eglob indicators correlations between 30‐min and each time windows were calculated to identify which time window yielded AD and Eglob values consistent with those of the 30‐min. Finally, the above partial correlation analyses were performed for each of the 11 time windows to validate the gF‐relevant AD and Eglob indicators across the different time windows.

## 3 Results

### 3.1 RAPM Score

According to the descriptive statistics, the average RAPM score was 25.24, with a standard deviation of 4.00. The minimum score was 16, and the maximum score was 36.

### 3.2 Resting‐State FCs of DLPFC Across Various Time Windows

**FIGURE 2:**
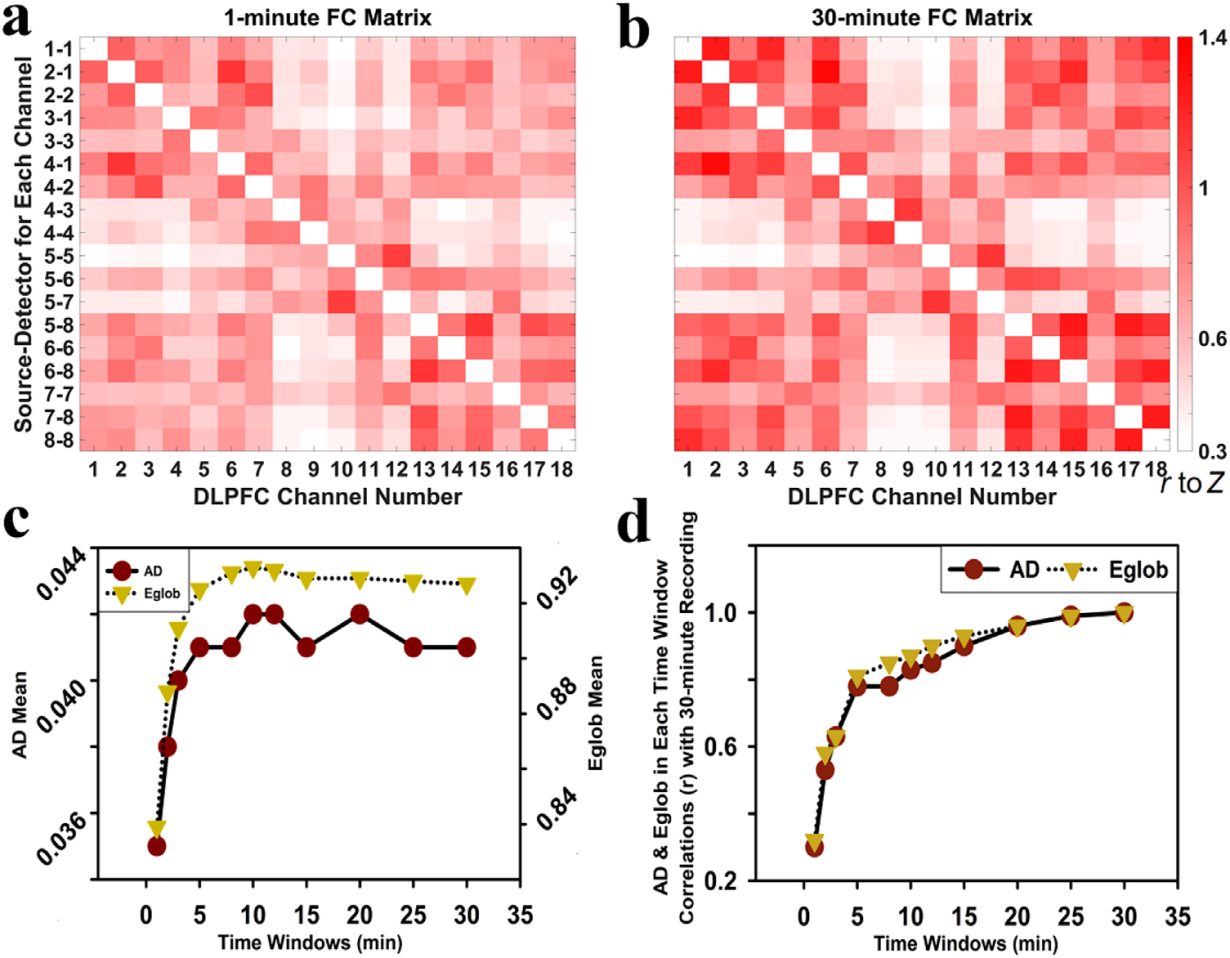
Resting‐state DLPFC channel FC correlation matrices averaged across 116 participants under different time windows: (a) the averaged FC matrix for the 1‐min time window; (b) the averaged FC matrix for the 30‐min time window; (c) the mean values of AD and Eglob across various time windows; (d) the values of AD and Eglob in each time window correlate with those of 30‐min recording.

The correlation matrices presented in the Figure 2a,b illustrate the resting‐state FCs between DLPFC channels, averaged across all 116 participants for the 1‐ and 30‐min time windows, respectively. The mean FCs of all DLPFC channels in the 1‐min FC matrix was 0.64, while the mean FC value in the 30‐min FC matrix was 0.74.

To determine the time window at which the AD and Eglob values of the DLPFC tend to stabilize, the mean values of AD and Eglob were calculated for 116 participants across 11 time windows (range from 1‐ to 30‐min, Figure 2c). The results showed that the mean values of both AD and Eglob began to stabilize after 5 min, with Eglob displaying a more consistent pattern of stability compared to AD. Furthermore, to verify the reproducibility of DLPFC graph‐theoretical indicators, the values of AD and Eglob of DLPFC under the 30‐min time window were subjected to partial correlation analyses (controlling for age and sex) with the corresponding values across the 11 time windows (range from 1‐ to 30‐min, Figure 2d). The analyses demonstrated t that after the 5‐min time window, both AD and Eglob showed stable consistency and strong correlations with the 30‐min time window AD (*r* = 0.78, *p* < 0.001) and Eglob (*r* = 0.81, *p* < 0.001).

### 3.3 Relationships Between DLPFC Graph Theoretical Indicators and gF

**FIGURE 3:**
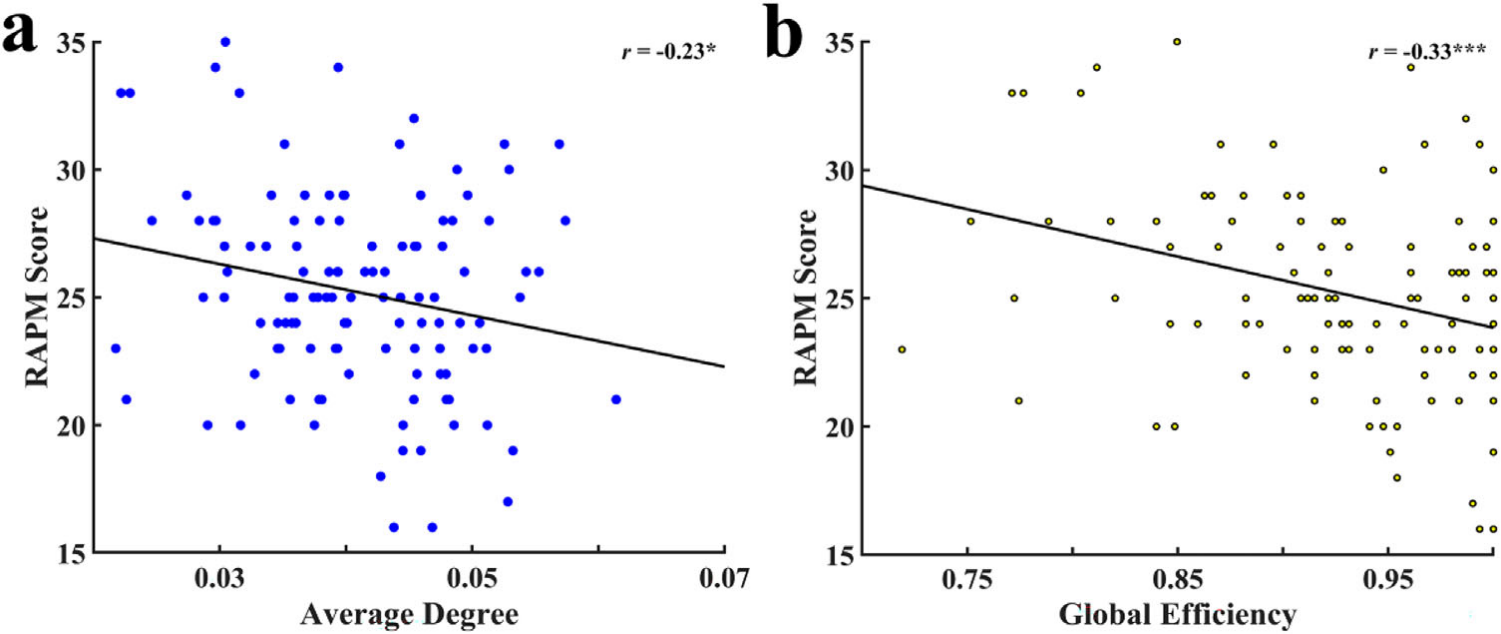
The relationship between graph theoretical indicators within 30‐min time window (AD and Eglob) and RAPM score. (a) Partial correlation between AD and RAPM score; (b) partial correlation between Eglob (using a threshold of 0.3 for Eglob binarization) and RAPM score.

Based on partial correlation analyses controlling for age and sex, significant negative relationships were observed between graph‐theoretical indicators within the 30‐min time window and the RAPM score. Specifically, the AD of the DLPFC in the resting‐state was negatively correlated with the RAPM score (*r* = −0.23, *p* < 0.05) (Figure 3a). When applying a binarization threshold of 0.3, the Eglob within the 30‐min time window also showed a significant negative correlation with the RAPM score (*r* = −0.33, *p* < 0.001) (Figure 3b). Furthermore, the correlation between Eglob and the RAPM score, evaluated across various binarization thresholds (ranging from 0.1 to 0.5 in increments of 0.1), consistently demonstrated negative relationships (Table 1).

**TABLE 1:** Partial correlations between Eglob within 30‐min time window and RAPM score across different binarization thresholds, controlling for age and sex.

	Statistic	Eglob_0.1	Eglob_0.2	Eglob_0.3	Eglob_0.4	Eglob_0.5
RAPM score	*r*	−0.36	−0.39	−0.33	−0.30	−0.30
	*p*	0.000	0.000	0.000	0.001	0.001

*Note*: RAPM refers to Raven's Advanced Progressive Matrices (Raven et al. 1998), “Eglob_0.1” refers to the global efficiency value (Latora and Marchiori 2001) at a binarization threshold of 0.1, and so forth.

### 3.4 Relationships Between DLPFC Graph Theoretical Indicators and gF Across Various Time Windows

**FIGURE 4:**
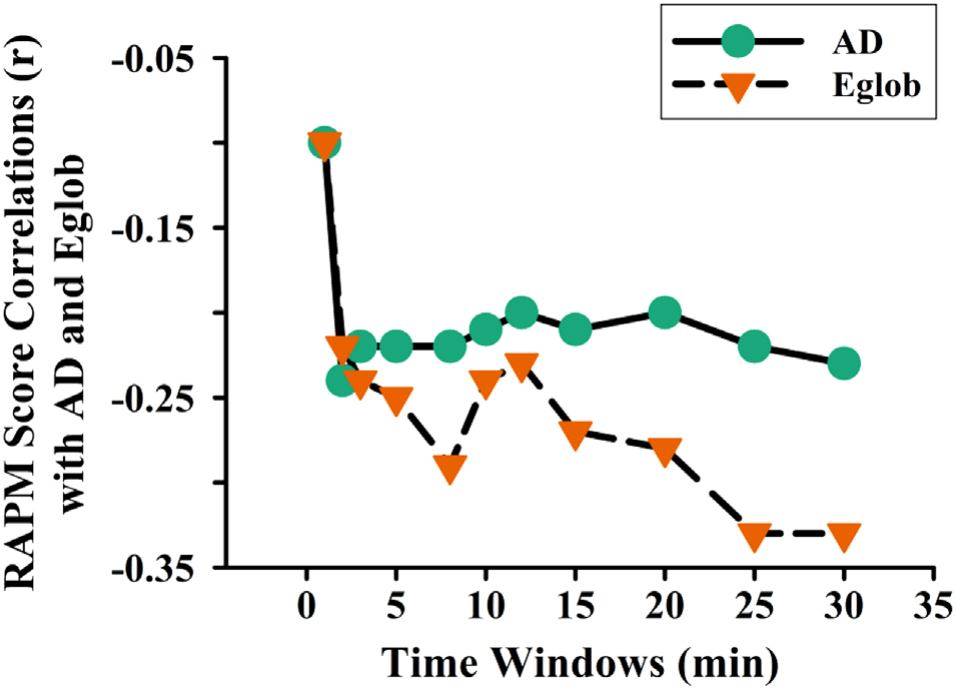
The effectiveness of graph‐theoretical indicators across various time windows: RAPM score correlations with AD and Eglob indicators.

To determine the suitable rs‐fNIRS acquisition time for revealing the relationship between DLPFC graph‐theoretical indicators and gF, partial correlation analyses were performed between the RAPM score and the AD, Eglob of the DLPFC in the resting‐state across 11 time windows, with age and sex as control variables (Figure 4). The results showed that the RAPM score exhibited a significant negative correlation with the AD of the DLPFC, which remained relatively stable around the 2‐min data collection period (*r* = −0.24, *p* < 0.05). Regarding the relationship between the RAPM score and Eglob of DLPFC, overall, the correlation became stronger as the time window increased. Within a shorter time window of approximately 2‐min, the RAPM score showed a significant negative correlation with the Eglob of DLPFC (*r* = −0.22, *p* < 0.05). For time windows of 15‐min or longer, the absolute correlation between the RAPM score and the Eglob of the DLPFC tended to increase with time, reaching *r* = −0.33 (*p* < 0.001) under the 30‐min time window.

## 4 Discussion

Our present study explored the graph‐theoretical mechanisms of gF in the DLPFC of college students using rs‐fNIRS technology. By analyzing gF from the perspective of connectionism, the two graph‐theoretical indicators, AD and Eglob, in rs‐fNIRS technology were identified as significantly negatively correlated with gF. The results also suggested that AD and Eglob could be suitable for rapid gF measurement within a 2‐min time window. These findings validate that rs‐fNIRS technology may aid in exploring the potential methods for gF assessment, revealing gF‐related individual differences in the specific topological features of resting‐state brain networks.

Based on the AD of FC matrices, the FC intensity gradually increased over time (Figure 2a,b), and the mean values of AD and Eglob stabilized after the 5‐min time window (Figure 2c). Moreover, the correlation results showed that after 5‐min data collected, AD and Eglob were strongly correlated with the 30‐min AD and Eglob (AD, *r* = 0.78, *p* < 0.001; Eglob, *r* = 0.81, *p* < 0.001) (Figure 2d), indicating that the AD and Eglob had similar effects in the 5‐ and 30‐min data collection periods. These findings suggest that a 5‐min data collection period was sufficient to reflect the stable characteristics of AD and Eglob. Consistent with previous research examining connectivity parameters from resting‐state data, it was concluded that a 5‐min fMRI scan is sufficient to obtain reliable estimates (Van Dijk et al. 2010). Therefore, when using AD and Eglob graph‐theoretical indicators, accurate results could be obtained with shorter data collection times, thereby enhancing data collection efficiency.

Furthermore, a correlation analysis between the rs‐fNIRS data and behavioral data was conducted, and the results revealed that the AD of the DLPFC in resting‐state was significantly negatively correlated with the RAPM score (Figure 3a). Consistent with previous studies which found people with higher levels of gF can solve cognitive problems with less resource allocation (Dunst et al. 2014; Lu et al. 2022), our results suggest that lower connectivity between resting‐state functions of the DLPFC is beneficial to gF performance. Specifically, individuals with higher levels of gF exhibit weaker internal functional network connectivity in the DLPFC and lower levels of information exchange in the corresponding brain regions during the resting‐state. Additionally, our results showed that Eglob of the DLPFC in the resting‐state was significantly negatively correlated with the RAPM score (Figure 3b and Table 1), and the correlation coefficient between Eglob and the RAPM score was higher than that between AD and the RAPM score. Based on the neural efficiency hypothesis, in the task state, the brain region related to the task is activated, while the irrelevant brain regions show reduced information communication to maximize efficiency (Dunst et al. 2014). In the resting‐state, the brain tends to reduce unnecessary information exchange and maintain a more relaxed state (Zakharov et al. 2020). This result is further supported by the concept of small‐world networks, which suggests that human brain networks are low‐energy consumption, high‐efficiency, and highly interconnected systems with extraordinary topological properties (Sporns and Zwi 2004; Stam et al. 2007).

However, since the participants did not perform tasks directly related to gF, caution is warranted to avoid overinterpreting the causal mechanisms underlying the relationship between the DLPFC FC and gF. Especially, it is important to emphasize the indirect, correlational nature of this relationship. There are confounding factors that could influence resting‐state network metrics, such as other cognitive functions (e.g., self‐control ability, as participants who were more patient and self‐controlled tended to exhibit higher‐quality resting‐state data with fewer motion artifacts [Siegel et al. 2017]) and physiological or psychological states (e.g., arousal levels, which can also affect resting‐state measures [Bijsterbosch et al. 2017]). Although some of these factors may themselves be components of, or overlap with gF (Panikratova et al. 2020; Santarnecchi et al. 2021), they raise the possibility that third‐variable effects could result in spurious correlations between gF and the DLPFC FC. Therefore, the current portable and objective rs‐fNIRS measurement of gF, should be considered an indirect reflection.

In addition to the reliability of AD and Eglob indicators, more attention is required regarding the temporal effectiveness of graph theoretical indicators related to gF. To address this, we investigated the gF‐relevant validity of two graph theoretical indicators across various time windows. The partial correlation between the RAPM score and the AD, Eglob of DLPFC showed that the data was relatively stable around the 2‐min, which is conducive to the rapid reflection of gF (Figure 4). However, to obtain a better reflection, the correlation between the RAPM score and the Eglob of the DLPFC could be calculated by collecting data for a longer period (more than 15 min). Additionally, the current results showed a continuing downward trend in the negative correlation between the DLPFC Eglob and the RAPM score within the 30‐min data collection window. While acquiring data for a brief 5‐min duration is sufficient for obtaining reliable rs‐fMRI and rs‐fNIRS data (Van Dijk et al. 2010), the duration of data collection significantly impacts the functional connectivity and graph‐theorical outcomes of brain networks (Birn et al. 2013; Liao et al. 2013). Longer data collection periods improve result test–retest reliability and robustness (Andellini et al. 2015), yet considering participant fatigue and time constraints is essential. Hence, further research is needed to elucidate the optimal rs‐fNIRS collection time for Eglob measurement effects on gF. Moreover, the absence of a short‐channel detectors in our study constitutes a limitation. By applying short‐channel regression methods, researchers can more effectively remove scalp and other superficial noise, thereby enhancing the reliability and effectiveness of fNIRS measurements.

## 5 Conclusion

This study explored the resting‐state DLPFC function network related to gF and preliminarily clarified the graph‐theoretical indicators for measuring gF using rs‐fNIRS technology. It found that the AD and Eglob of DLPFC in the resting‐state were significantly negatively correlated with the RAPM score, with Eglob of the DLPFC emerging as a more suitable graph‐theoretical indicator for measuring gF. Additionally, the study provided insights into the appropriate and effective data collection time window for measuring gF, thereby advancing the development of portable and objective gF assessment using rs‐fNIRS.

## Author Contributions

**Yuemeng Wang**: conceptualization, data curation, formal analysis, visualization, writing – original draft, writing – review and editing. **Zhencai Chen**: conceptualization, methodology, funding acquisition, project administration, writing – review and editing. **Ziqi Cai**: investigation, formal analysis, writing – original draft. **Wenqun Ao**: investigation, formal analysis, writing – original draft. **Qi Li**: investigation. **Ming Xu**: investigation. **Suyun Zhou**: investigation.

## Conflicts of Interest

The authors declare no conflicts of interest.

### Peer Review

The peer review history for this article is available at https://publons.com/publon/10.1002/brb3.70386.

## Data Availability

The data that support the findings of this study are available on reasonable request from the corresponding author. The data are not publicly available due to privacy or ethical restrictions.
